# What to Prefer in Patients with Multibracket Appliances? Digital vs. Conventional Full-Arch Impressions—A Reference Aid-Based In Vivo Study

**DOI:** 10.3390/jcm12093071

**Published:** 2023-04-23

**Authors:** Niko Christian Bock, Katharina Klaus, Moritz Maximilian Liebel, Sabine Ruf, Bernd Wöstmann, Maximiliane Amelie Schlenz

**Affiliations:** 1 Department of Orthodontics, Dental Clinic, Justus Liebig University, Schlangenzahl 14, 35392 Giessen, Germany; katharina.klaus@dentist.med.uni-giessen.de (K.K.); moritz.m.liebel@med.uni-giessen.de (M.M.L.); sabine.ruf@dentist.med.uni-giessen.de (S.R.); 2 Department of Prosthodontics, Dental Clinic, Justus Liebig University, Schlangenzahl 14, 35392 Giessen, Germany; bernd.woestmann@dentist.med.uni-giessen.de

**Keywords:** full-arch impression, multibracket appliance, intraoral scanner, digital dentistry, alginate, accuracy, precision, trueness, clinical study, reference

## Abstract

This study aimed to investigate the transfer accuracy and required time for digital full-arch impressions obtained from intraoral scanners (IOSs) versus conventional alginate impressions (CAIs) in patients with multibracket appliances (MBA). Thirty patients with buccal MBAs (metal brackets, archwire removed) were examined using an established reference aid method. Impression-taking using four IOSs (Primescan, Trios 4, Medit i700, Emerald S) and one CAI with subsequent plaster casting were conducted. One-hundred-twenty (n = 30 × 4) scans were analyzed with 3D software (GOM Inspect) and 30 (n = 30 × 1) casts were assessed using a coordinate measurement machine. Six distances and six angles were measured and compared to the reference aid values (ANOVA; *p* < 0.05). Except for the intermolar distance, transfer accuracy was significantly higher with IOSs than with CAIs (*p* < 0.05). No such difference was found regarding the six angles. In patients with MBAs, digital impression-taking using IOSs can be recommended. For all measured variables except one, the transfer accuracy of IOSs was better than or at least equivalent to the data from CAIs. In addition, significantly (*p* < 0.001) less time was necessary for all IOSs in comparison to CAIs plus plaster casting.

## 1. Introduction

Conventional alginate impressions (CAIs) and subsequent conventional plaster model casting (CPC) have been the gold standard in orthodontics for decades. While CAIs have been able to serve most purposes, situations with undercuts can be expected to result in severe deformations or tear-out effects, which have been demonstrated in aged dentitions exhibiting multiple undercuts [1]. Therefore, situations with undercut-causing attachments on tooth surfaces, such as a fixed multibracket appliance (MBA), might also result in an incorrect display of the intraoral or dental condition, which might have severe consequences, for example, when preparing a splint for orthognathic surgery [1].

Meanwhile, intraoral scanners (IOSs) are widely used in orthodontics [2,3,4,5,6,7,8,9] and several advantages of digital impressions in favor to CAI and CPC have been proven, including higher patient comfort, lower amount of waste and less necessary storage space, as well as higher accuracy [10,11,12,13]. However, the accuracy of digital impression-taking with an IOS differs a lot when comparing dental arches with and without a bonded MBA. While two prior in vitro studies determined no clinically relevant effect of MBAs on the impression accuracy [14,15], more recent investigations described a distinct reduction in the transfer accuracy for full-arch impressions [16,17,18]. The effect, however, is still acceptable when compared to the data determined for CAIs [18].

Regarding the amount of time needed to obtain a full-arch impression, several studies have been published [11,13,18,19,20]. While CAIs might demand the lowest amount of chairside time for the impression itself, the chairside time to remove and reinsert the archwire—which is recommended for improved impression quality and reduced patient discomfort—as well as the duration of the subsequent obligatory CPC process in the dental laboratory need to be considered. Therefore, CAIs have been shown to be less advantageous or equal (at best) compared to digital impressions [11,12,18,20]. Nevertheless, the presence of an MBA has been shown to increase the necessary amount of time needed for IOS in comparison to situations with natural teeth only [18,19].

The majority of studies investigating the accuracy of impression-taking in models or patients with MBAs used a best-fit method for comparison of digital and conventional impression techniques [19]. However, those investigations could only detect differences between the applied impression techniques themselves, but are not able to determine the accuracy (trueness) of the impression compared to the actual in vitro or in vivo situation [21,22]. For the latter, a reference is mandatory [23,24,25]. Furthermore, the best-fit methods use an approximate calculation, resulting in a minimization of mesh errors, which leads to an underestimate of the actual transfer error across the complete arch [21,22]. Overall, the amount of reliable data on transfer accuracy in terms of trueness and precision, as well as the required time for digital full-arch impressions by IOSs in orthodontic patients with MBAs, are scarce, particularly in comparison to the current gold standard, CAIs.

Therefore, in a previous in vitro study, the authors investigated the established reference-aid based methodology in different model settings, with and without MBA, using five IOSs for digital and one CAI for conventional impression-taking [18]. Even though the results of the laboratory study were promising, the data from a clinical setting might differ a lot from the in vitro data due to patient movement, soft tissue mobility, saliva flow, and anatomically-limited space for intraoral scanning tip or impression tray [26,27,28]. Thus, this clinical study aims to assess the transfer accuracy of full-arch impressions in orthodontic patients with MBAs. In addition, the necessary amount of time was analyzed.

The first null hypothesis was that no significant difference exists regarding the transfer accuracy of digital (IOS) and conventional (CAI) full-arch impressions in orthodontic patients with MBAs. The second null hypothesis was that no significant difference exists between the two procedures in terms of the necessary amount of time for impression-taking and additional processing until a model for diagnostic purposes is available.

## 2. Materials and Methods

### 2.1. Patient Sample

After ethical approval (ref. no. 71/19, Medical Faculty, Justus Liebig University Giessen) and study registration (DRKS00028304), the recruitment of orthodontic fixed appliance patients started at the Department of Orthodontics, Justus Liebig University Giessen. Based on the results of a previous in vitro study [18], a sample size calculation revealed a total of 30 patients to be sufficient to answer the research questions.

The following inclusion and exclusion criteria were applied.

Inclusion criteria:

- Age ≥ 16 years;
- MBA treatment in the lower jaw ongoing for at least three months;
- Buccal metal brackets Tip-Edge PLUS Stainless Steel Brackets (TP Orthodontics Inc., La Porte, IN, USA) on all lower teeth except molars;
- Metal bands Unitek Victory Series First Molar Bands (3 M, St. Paul, MN, USA) cemented on the lower-right and left first molar adjacent to the premolars with glass ionomer luting cement Ketac Cem (3 M, St. Paul, MN, USA).
- Exclusion criteria:
- Lower teeth with restorations in metal color;
- Spaces ≥ 2 mm in case of aplasia or extraction.

Recruitment took approximately ten weeks (27 January–4 April 2022). Informed consent was obtained from all participants, and the study was performed in accordance with the Declaration of Helsinki. Study participation took place during a regular orthodontic check-up visit. The orthodontic archwire was removed from the brackets immediately before the start of the study.

### 2.2. Reference Aid and Reference Dataset

**Figure 1:**
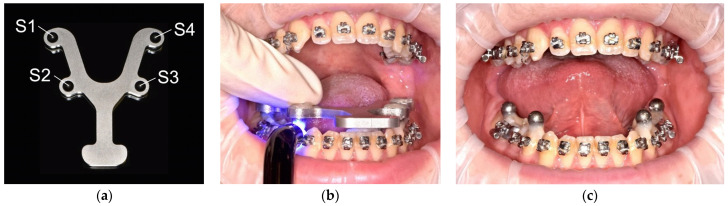
Reference aid with the four high-precision bearing spheres (S1–S4) (**a**), intraoral placement of reference spheres (S1–S4) with (**b**) and without (**c**) reference aid.

As in previous studies investigating the transfer accuracy of full-arch impressions [24,25,29], a magnetic reference aid was used to position four high-precision bearing spheres (1.3505 100Cr6 DIN5401 [30], TIS GmbH, Gauting, Germany) with a roundness of 5000 ± 5.63 μm [31] on the occlusal surface of the lower first premolars and second molars (Figure 1a–c). The spheres were reversibly bonded to the respective surfaces using flowable composite (Grandio Flow, Voco, Cuxhaven, Germany). This procedure has been shown to be reproducible (precision < 10 μm) [29]. The reference dataset was obtained by performing multiple measurements (*n* = 10) of the reference aid with the four spheres inserted using a coordinate measurement machine (CMM, Thome Präzision GmbH, Messel, Germany) and the corresponding 3D software (X4 V10 GA × 64, Metrologic Group, Meylan, France). Afterwards, the mean values of the sphere positions were calculated; the reference dataset was kept as initial graphics exchange specification (IGES) format.

### 2.3. Impression-Taking

In each patient, the clinical part of the study started with the digital impression-taking. IOSs were calibrated before each scanning, if provided, according to the manufacturer’s instructions [32]. The following sequence of IOSs were applied:

1. Primescan (“PRI”, version 5.1.3, Dentsply Sirona, Bensheim, Germany);
2. Trios 4 POD wireless (“TIO“, version 21.2.0, 3Shape, Copenhagen, Denmark);
3. Planmeca Emerald S (“EME”, version 6.2.1.25, Planmeca, Helsinki, Finland);
4. Medit i700 (“MED”, version 1.7.4, Medit, Seoul, Republic of Korea).

The same scanning path, occlusal surfaces–lingual surfaces–buccal surfaces, was conducted to ensure comparable data acquisition [33]. Afterwards, the scan datasets were exported from the IOSs as a standard tessellation language (STL) file. The CAI was taken last with an alginate (Cavex Orthotrace, batch no. 210204, Cavex Holland, Haarlem, The Netherlands) being prepared and mixed in a standardized way (Migma 200, Mikrona Technologie, Schlieren, Switzerland) according to the manufacturer’s instructions. The impression was taken with a full-arch metal tray (Ehricke stainless steel, Orbis Dental, Germany), where a thin layer of adhesive (Fix Tray Adhesive for Alginate Impression Materials, Dentsply DeTrey GmbH, Konstanz, Germany) had been applied. The impression tray was kept in the oral cavity for a setting time of one minute. After disinfection (MD520, Dürr Dental, Kornwestheim, Germany) and storage in a moist environment for a maximum of 15 min, the CAI was cast with type IV dental stone (Fujirock EP, GC Europe, Leuven, Belgium) using a base former [34]. Thereafter, the plaster casts were stored under laboratory conditions for a minimum of seven days before performing any measurement.

**Figure 2:**
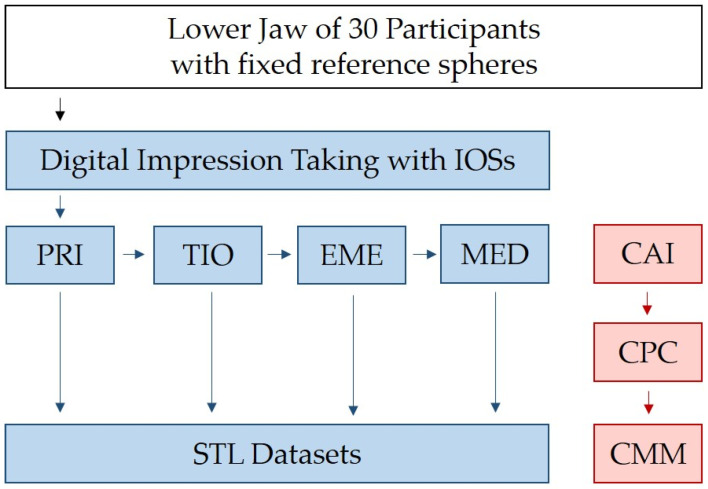
Flow scheme of the investigation (IOSs = intraoral scanners, PRI = Primescan, TIO = Trios 4, EME = Emerald S, MED = Medit i700, CAI = conventional alginate impression, CPC = conventional plaster model casting, STL = standard tessellation language, CMM = coordinate measuring machine).

The assessment of the time necessary for both digital impressions as well as CAIs plus CPC was performed with an electronic clock. The time between CAI and CPC as well as the plaster hardening time were not counted. Figure 2 displays a flow scheme of the investigation.

### 2.4. Analysis of Transfer Accuracy

**Figure 3:**
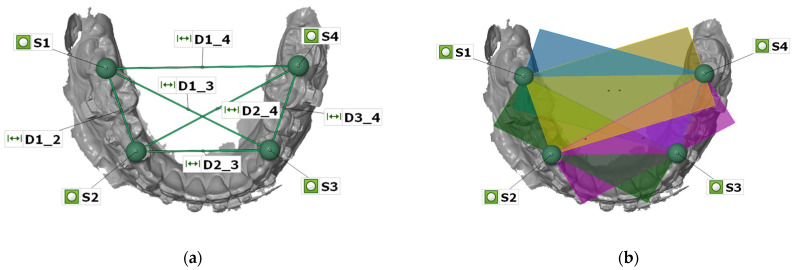
Measurement of linear distances (D1_2, D1_3, D1_4, D2_3, D2_4, D3_4) between the centers of the four spheres (S1–S4) (**a**), constructed planes (P1–P4) for measurement of the six angles (**b**), displayed in GOM Inspect analysis software.

All STL datasets generated by the IOSs were imported to the 3D analysis software GOM Inspect (version 2020, GOM GmbH, Braunschweig, Germany) as actual data and the reference dataset as CAD data. The actual datasets of the IOSs were imported as linked point clouds; therefore, the first four spheres were constructed in the position of the spheres using fitting elements (Gauß best-fit, 3 Sigma). Subsequently, the linear distances (D1_2, D1_3, D1_4, D2_3, D2_4, D3_4) displayed in Figure 3a were measured between the centers of the spheres for each IOS dataset, and the deviations from the reference dataset were calculated. For the angular measurements, four planes—each through the centers of three spheres—were constructed (P1: S1–S2–S3, green; P2: S1–S2–S4, yellow; P3: S1–S3–S4, blue; P4: S2–S3–S4, purple, Figure 3b), and six angles were measured between the planes (A1_2, A1_3, A1_4, A2_3, A2_4, A3_4) for each IOS dataset. According to the linear distances, the deviations from the reference dataset were calculated.

The assessment of the CAI data and the plaster cast data was performed with the CMM, using a specifically-programmed mode of operation which automatically allowed the surface sampling of the spheres with the measuring sensor. Subsequently, the center of each sphere was calculated, and the distances between the centers of the spheres were measured using the corresponding CMM software(X4 V10 GA × 64, Metrologic Group, Meylan, France).

SPSS Statistics (version 26, IBM, Armonk, NY, USA) was used for the statistical analysis. A two- or three-way ANOVA was performed. Due to the heterogeneity of variances, the SPSS procedure GENLINMIXED was applied [35]. For a detailed evaluation of the different groups and distances, one-way ANOVAs with impression technique as six-step factor were performed. The analyses were carried out as non-parametric Kruskal–Wallis tests due to extreme outliers. A Bonferroni–Holm correction was applied and the level of significance was *p* < 0.05.

Accuracy in terms of trueness (mean deviations between the impressions, the resulting models, and the reference aid) and precision (standard deviations) was described according to ISO 5725-1 [36].

No intra- or interrater reliability was determined, as no manual measurements were taken and only one investigator (M.M.L.) was involved, who was trained and calibrated as to all impression techniques and measurement methods in advance of this study.

## 3. Results

**Figure 4:**
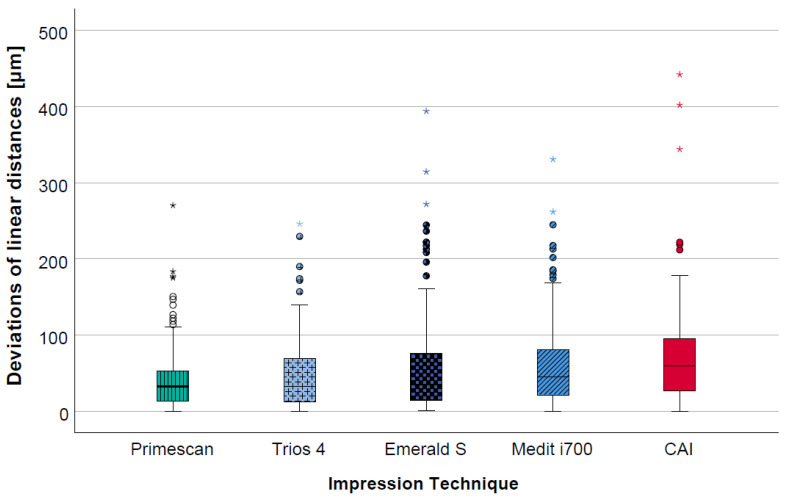
Boxplot diagram displaying the pooled data of the deviations of the linear distances (D1_2, D1_3, D1_4, D2_3, D2_4, D3_4) for the different impression techniques (CAI = conventional alginate impression); outliers (^O^), extreme values (*).

The calculated pooled deviations of the linear distances (D1_2, D1_3, D1_4, D2_3, D2_4, D3_4) between the reference dataset and the data from the digital and the conventional impressions showed lower deviations for all IOSs (range: 42 ± 41 µm to 60 ± 56 µm) in comparison to the CAI (71 ± 65 µm). Considering the different IOSs, slightly better results were seen for PRI (42 ± 41 µm) and TIO (47 ± 44 µm) compared to EME (57 ± 64 µm) and MED (60 ± 56 µm) (Table 1 and Figure 4).

Evaluating the deviations for each separate linear distance, ranges of 20 ± 17 µm to 125 ± 70 µm and 58 ± 40 µm to 83 ± 81 µm (in percentages: 0.08 ± 0.07% to 0.27 ± 0.22%, and 0.17 ± 0.12% to 0.30 ± 0.35%) were observed for IOSs and CAI, respectively. For the short distances within a quadrant (D1_2 and D3_4), a significant difference between the four IOSs and the CAI was noted (*p* < 0.05). Long intermolar distances (D1_4) showed a higher deviation between PRI/TIO and EME/MED, although no significant differences were found, neither between the IOSs, nor between the IOSs and CAI.

**Figure 5:**
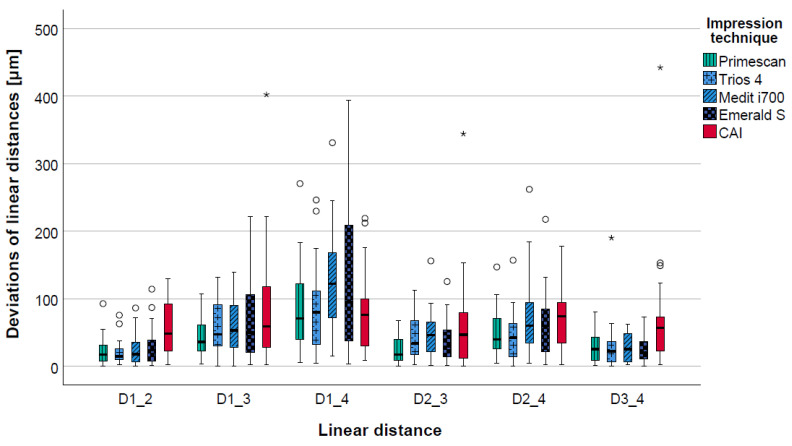
Boxplot diagram displaying the deviations of the linear distances (D1_2, D1_3, D1_4, D2_3, D2_4, D3_4) for the different impression techniques (CAI = conventional alginate impression); outliers (^O^), extreme values (*).

The detailed data for trueness and precision of each separate linear distance (D1_2, D1_3, D1_4, D2_3, D2_4, D3_4) are given in Table 1 and Figure 5, as well as Appendix A (Table A1), which contains the respective *p*-values.

**Figure 6:**
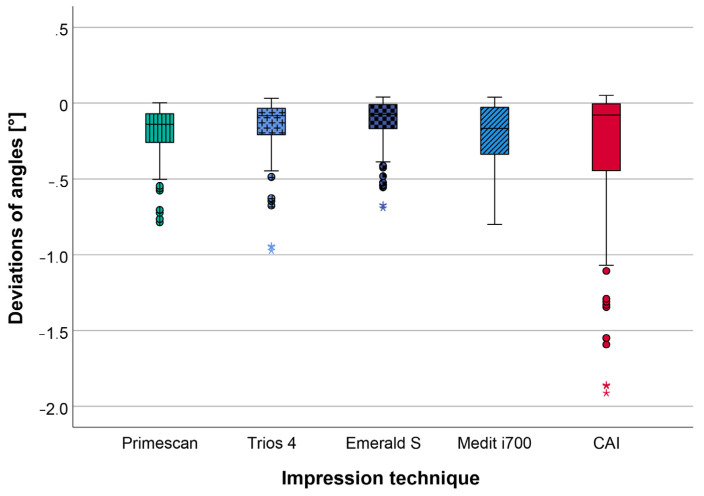
Boxplot diagram displaying the pooled data of the deviations of the angles (A1_2, A1_3, A1_4, A2_3, A2_4, A3_4) for the different impression techniques (CAI = conventional alginate impression); outliers (^O^), extreme values (*).

The calculated pooled deviations of the angles (A1_2, A1_3, A1_4, A2_3, A2_4, A3_4) between the reference dataset and the data of the impressions (Table 2 and Figure 6) also revealed lower deviations for all IOSs (range: 0.12 ± 0.15° to 0.19 ± 0.20°) in comparison to the CAI (0.28 ± 0.43°). Looking at the different IOSs, slightly better results were seen for TIO (0.14 ± 0.18°) and EME (0.12 ± 0.15°) compared to PRI (0.19 ± 0.17°) and MED (0.19 ± 0.20°). Overall, the highest scattering in data was observed for CAI, resulting in the lowest precision for all investigated impression techniques.

**Figure 7:**
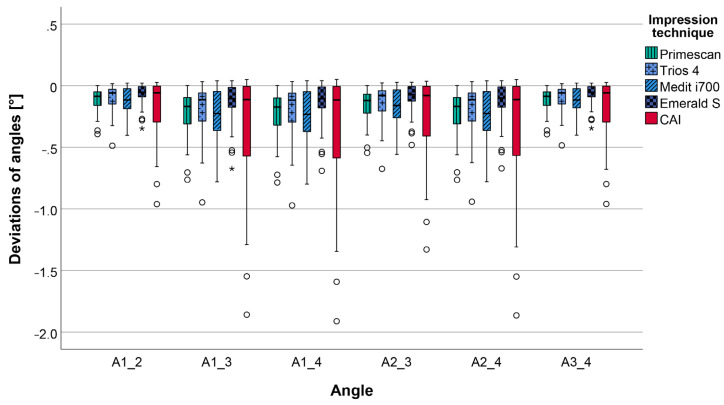
Boxplot diagram displaying the deviations of the angles (A1_2, A1_3, A1_4, A2_3, A2_4, A3_4) for the different impression techniques (CAI = conventional alginate impression); outliers (^O^), extreme values (*).

Considering the deviations of each separate angle, ranges of 0.08 ± 0.10° to 0.25 ± 0.24° and 0.18 ± 0.27° to 0.37 ± 0.53° were seen for IOSs and CAI, respectively. No significant differences (*p* ≤ 0.05) existed, neither between any IOSs and CAI, nor between different IOSs. The respective data are given in Table 2 and Figure 7 as well as Appendix A (Table A2), which contains the respective *p*-values.

Nevertheless, the primary null hypothesis, that no significant difference existed between the transfer accuracy of digital (IOSs) and conventional (CAI) full-arch impressions in orthodontic patients with MBAs, had to be rejected.

**Figure 8:**
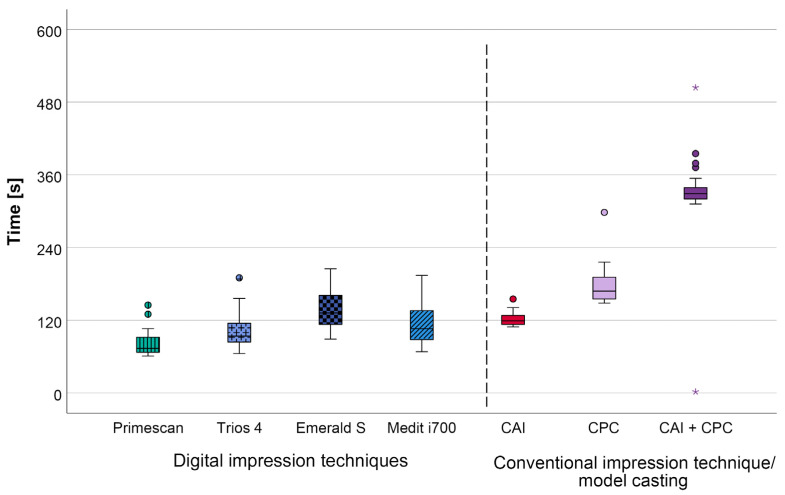
Required amount of time [s] for impression-taking using the different impression techniques (CAI = conventional alginate impression, CPC = conventional plaster model casting, CAI + CPC = sum of conventional alginate impression and conventional plaster model casting without setting time); outliers (^O^), extreme values (*).

Examining the amount of time necessary for both IOSs as well as CAIs plus CPC, significantly lower values (*p* < 0.001) were measured for all IOSs (range: 80 ± 20 s to 136 ± 31 s) than for CAIs plus CPC (349 ± 30 s). Comparing the IOSs to chairside CAI time still reveals lower values for all IOSs but one (Figure 8 and Table 3).

Therefore, the second null hypothesis, that no significant differences existed between the two procedures in terms of the time necessary for impression-taking and additional processing until a model for diagnostic purposes was available, had to be rejected.

## 4. Discussion

Although the use of digital full-arch impressions obtained by IOSs shows an ever -ncreasing popularity in orthodontics, the number of published studies on the clinical performance with MBAs in situ is low [37]. Distinct conclusions based on reliable data and reference systems regarding accuracy in terms of trueness and precision are missing.

Therefore, the aim of the present clinical study was to perform an evaluation of the transfer accuracy in terms of trueness and precision of digital and conventional full-arch impressions in patients with MBAs without archwires. In contrast to most other published investigations [14,15,16,19], a well-established reference aid-based method was applied.

The patient sample was rather homogenous, as all participants were recruited from the same department applying strict inclusion/exclusion criteria and had the same kind of MBA in situ (identical brackets and metal bands). In addition, all impressions were taken in a standardized way, by the same operator who also performed all laboratory procedures and measurements. Thus, a high level of standardization can be assumed. However, it can be discussed that the same scanning path was applied for all IOSs, even though some manufacturers described another one in their instructions for use. For better comparison of scan data, we decided to use the established scanning path that has been well-investigated and -described in the literature as clinically accepted [33].

Due to the limited amount of data available in the literature, a comparison of the present results to other data is difficult.

Looking at the accuracy of the IOSs regarding the linear distances in general, all measured deviations were ≤0.4 mm including maximum outliers (mean values: 0.04–0.06 mm); for CAI, the respective maximum value was 0.5 mm (mean value: 0.07 mm). In addition, while digital impressions generally revealed better values than CAI, this advantage decreased with increasing length of the respective distances, with CAI exhibiting the best transfer accuracy for the longest assessed distance. Comparing the values determined for the different IOSs, almost no significant differences were observed. Nevertheless, for most orthodontic purposes, all measured deviations seemed to be without clinical relevance.

One clinical trial in patients with MBAs in situ used a quite different method to assess and compare digital and conventional impressions, but still determined both procedures to be similarly accurate [38]. Another clinical full-arch assessment—in patients without MBAs, however—obtained slightly higher deviations (range: 51 ± 38 µm to 108 ± 99 µm) for comparable linear distances to the present study (range: 42 ± 41 µm to 108.0 ± 60.6 µm) [39]. Slightly lower deviations between conventional and digital models (range: 15 ± 13 µm to 32 ± 22 µm) were described for a mixed dentition patient sample with natural teeth [40]. It should be kept in mind, however, that the IOSs used in the current investigations were more advanced. In addition, none of the other clinical studies applied a fully stable external reference for comparison, which might affect the reliability of the data.

A recently-published in vivo study using the same reference methodology and IOSs (PRI, TIO, EME and MED) as investigated in this study revealed lower transfer accuracy for PRI and TIO in patients without MBA than in the current study with MBA [41]. However, considering the fact that manufacturers of IOSs do not disclose their algorithms, it can only be hypothesized that, for some IOSs, the MBA might be helpful for superimposition, resulting in higher transfer accuracy. The latter could be even more the case with archwires in place. In the present study, they were removed because CAI impression-taking with MBAs is generally recommended without archwires to reduce undercuts and the degree of patient discomfort.

Comparing data from this clinical study to the previously-published in vitro findings [18], PRI obtained even better results in vivo than in vitro with the same hardware and software. However, for TIO and EME with the same hardware but an updated software version, higher deviations for linear distances were shown in vivo. Data from MED are difficult to compare because different hardware and software generations were investigated (Medit i500 in vitro and Medit i700 in vivo). Concerning CAI, better results could be shown in patients than in the model situation. This can be explained by the alginate material, which requires a moist environment for best performance.

Looking at the deviations of the angular measurements, no comparable clinical data have been published so far. Nevertheless, data from an in vitro investigation where the same method as in the current study had been applied showed slightly higher deviations (range: 0.2 ± 0.4 to 0.9 ± 0.8° compared to 0.1 ± 0.2 to 0.3 ± 0.4°) and a significant difference (*p* ≤ 0.001) between the digital and conventional procedure, which was not the case in the present investigation [18,24]. The minor differences between the two studies, and possible reasons might be the different setting—in vitro vs. in vivo—as well as the more advanced IOSs and IOSs’ software.

The amount of time necessary for a full-arch impression was found to be significantly lower for all IOSs compared to CAI plus CPC (*p* < 0.001); interestingly, one IOS (PRI) also exhibited a significant difference when compared to all other IOSs, which did not differ from each other (80 ± 20 s vs. range: 102 ± 29 s to 136 ± 31 s; CAI: 122 ± 11 s, CAI + CPC: 349 ± 40 s).

The majority of published studies dealing with digital and conventional full-arch impressions only considered teeth without any fixed orthodontic appliances [10,11,12,13,20] and determined equal [11,12] or significantly shorter chairside time for CAI [20]. However, the processing time in the laboratory is subsequently needed after taking a CAI to obtain a proper model for diagnostic or any other purposes, and should therefore be considered in addition to the chairside time, resulting in the conventional procedure being disadvantageous [20], as it is in concordance with the results of the present investigation. The time advantage of the IOSs could potentially increase even more if the archwires would not need to be removed. The latter will be investigated in a future study.

Other factors influencing the required scanning time might be the processing power of the available hard- and software as well as the orthodontic appliance, as this might act as a type of guiding tool for the IOS, and accelerate the processing of the scanned images.

Regarding limitations, the fact that only the occlusal-bonded reference spheres, and no further occlusal, buccal or lingual surfaces were included for measurements, certainly needs to be considered. This was due to the defined and stable reference structure, which has proven to be reliable to compare the measurements [24,25]. As no such reference structure has been described for buccal or lingual tooth surfaces yet, we decided to take advantage of the known system. In addition, the fact that only the mandible was used for the placement of the reference aid and the measurements has to be considered as a limitation. Unfortunately, the current version of the reference aid cannot be used in the maxilla for reasons of safety. Furthermore, no impressions of the natural teeth (without MBA in situ) as further reference dataset could be determined in this study, as the participants were still undergoing active orthodontic treatment.

Nevertheless, the present data showed that digital impressions can be recommended for patients with MBAs (no archwire) in situ. While the clinical setting of the present study was designed with the removal of the orthodontic archwire before impression-taking, it might be worthwhile to re-investigate in a clinical setting where the archwire remains in situ.

## 5. Conclusions

The transfer accuracy in terms of trueness and precision with IOSs was better than or at least equivalent to the data from CAIs for all measured variables, except the longest distance. In addition, significantly less time was needed for a digital full-arch impression compared to a CAI, and both null hypotheses had to be rejected. Therefore, digital full-arch impression-taking using IOSs, which the participants seemed to favor over CAI, can be recommended in patients with MBAs (without archwire).

## Figures and Tables

**Table 1:** Deviations of the linear distances (pooled data as well as D1_2, D1_3, D1_4, D2_3, D2_4, D3_4) for the different impression techniques (PRI = Primescan, TIO = Trios 4, EME = Emerald S, MED = Medit i700, CAI = conventional alginate impression), shown as mean for trueness [µm] and standard deviation (SD) for precision [µm], according to the International Organization for Standardization (ISO) 5725-135 [36].

Linear Distance	Mean (Trueness) [µm] ± SD (Precision) [µm]				
	PRI	TIO	EME	MED	CAI
Pooled data	42 ± 41	47 ± 44	57 ± 64	60 ± 56	71 ± 65
D1_2	23 ± 20	20 ± 17	29 ± 27	25 ± 23	58 ± 40
D1_3	43 ± 29	59 ± 39	67 ± 59	60 ± 38	83 ± 81
D1_4	88 ± 62	84 ± 62	123 ± 101	125 ± 70	80 ± 59
D2_3	26 ± 20	43 ± 32	37 ± 31	48 ± 32	62 ± 69
D2_4	48 ± 34	45 ± 35	59 ± 48	73 ± 62	74 ± 52
D3_4	27 ± 22	30 ± 36	24 ± 18	28 ± 20	69 ± 81

**Table 2:** Deviations of the angles (pooled data as well as A1_2, A1_3, A1_4, A2_3, A2_4, A3_4) for the different impression techniques (PRI = Primescan, TIO = Trios 4, EME = Emerald S, MED = Medit i700, CAI = conventional alginate impression), shown as mean for trueness [µm] and standard deviation (SD) for precision [µm], according to the International Organization for Standardization (ISO) 5725-135 [36].

Angle	Mean (Trueness) [°] ± SD (Precision) [°]				
	PRI	TIO	EME	MED	CAI
Pooled data	0.19 ± 0.17	0.14 ± 0.18	0.12 ± 0.15	0.19 ± 0.20	0.28 ± 0.43
A1_2	0.12 ± 0.10	0.09 ± 0.11	0.08 ± 0.10	0.13 ± 0.12	0.18 ± 0.27
A1_3	0.23 ± 0.19	0.18 ± 0.21	0.15 ± 0.19	0.24 ± 0.23	0.36 ± 0.52
A1_4	0.24 ± 0.20	0.19 ± 0.22	0.15 ± 0.19	0.25 ± 0.24	0.37 ± 0.53
A2_3	0.17 ± 0.14	0.13 ± 0.15	0.11 ± 0.13	0.17 ± 0.17	0.25 ± 0.37
A2_4	0.23 ± 0.19	0.18 ± 0.21	0.15 ± 0.19	0.24 ± 0.23	0.35 ± 0.52
A3_4	0.12 ± 0.10	0.09 ± 0.11	0.08 ± 0.10	0.12 ± 0.12	0.18 ± 0.27

**Table 3:** Required amount of time [s] until a model for diagnostic purposes was available for the different impression techniques (PRI = Primescan, TIO = Trios 4, EME = Emerald S, MED = Medit i700, CAI = conventional alginate impression, CPC = conventional plaster model casting). In addition, the *p*-values of the pairwise comparison are given.

Impression Technique	PRI 80 ± 20 s	TIO 102 ± 29 s	EME 136 ± 31 s	MED 116 ± 36 s
TIO 102 ± 29 s	0.004	-	-	-
EME 136 ± 31 s	<0.001	0.063	-	-
MED 116 ± 36 s	0.024	0.202	0.057	-
CAI 122 ± 11 s	<0.001	0.004	0.052	0.410
CAI + CPC 349 ± 40 s	<0.001	<0.001	<0.001	<0.001

## Data Availability

The datasets of this article are available from the corresponding author on a reasonable request.

## Appendix A

**Table A1:** *p*-values of the pairwise comparison of the different impression techniques (PRI = Primescan, TIO = Trios 4, EME = Emerald S, MED = Medit i700, CAI = conventional alginate impression) in terms of deviations from the reference data set (linear distances D1_2, D1_3, D1_4, D2_3, D2_4, D3_4).

Linear Distance	Impression Technique	PRI	TIO	EME	MED
D1_2	TIO	1.000	-	-	-
	EME	1.000	0.605	-	-
	MED	1.000	1.000	1.000	-
	CAI	<0.001	0.009	0.007	0.001
D1_3	TIO	0.458	-	-	-
	EME	0.401	1.000	-	-
	MED	0.401	1.000	1.000	-
	CAI	0.090	0.795	0.795	1.000
D1_4	TIO	1.000	-	-	-
	EME	0.491	0.410	-	-
	MED	0.216	0.136	1.000	-
	CAI	1.000	1.000	0.288	0.064
D2_3	TIO	0.099	-	-	-
	EME	0.504	0.003	-	-
	MED	0.013	0.383	0.035	-
	CAI	0.048	0.137	<0.001	0.018
D2_4	TIO	1.000	-	-	-
	EME	1.000	1.000	-	-
	MED	0.321	0.212	1.000	-
	CAI	0.164	0.092	1.000	1.000
D3_4	TIO	1.000	-	-	-
	EME	1.000	1.000	-	-
	MED	1.000	1.000	1.000	-
	CAI	0.049	0.087	0.027	0.049

**Table A2:** *p*-values of the pairwise comparison of the different impression techniques (PRI = Primescan, TIO = Trios 4, EME = Emerald S, MED = Medit i700, CAI = conventional alginate impression) in terms of deviations from the reference data set (angles A1_2, A1_3, A1_4, A2_3, A2_4, A3_4).

Angles	Impression Technique	PRI	TIO	EME	MED
A1_2	TIO	1.000	-	-	-
	EME	0.766	1.000	-	-
	MED	1.000	1.000	0.766	-
	CAI	1.000	0.766	0.393	1.000
A1_3	TIO	1.000	-	-	-
	EME	0.758	1.000	-	-
	MED	1.000	1.000	0.758	-
	CAI	1.000	0.758	0.388	1.000
A1_4	TIO	1.000	-	-	-
	EME	0.669	1.000	-	-
	MED	1.000	1.000	0.669	-
	CAI	1.000	0.669	0.359	1.000
A2_3	TIO	1.000	-	-	-
	EME	0.668	1.000	-	-
	MED	1.000	1.000	0.668	-
	CAI	1.000	0.668	0.356	1.000
A2_4	TIO	1.000	-	-	-
	EME	0.657	1.000	-	-
	MED	1.000	1.000	0.657	-
	CAI	1.000	0.657	0.373	1.000
A3_4	TIO	1.000	-	-	-
	EME	0.731	1.000	-	-
	MED	1.000	1.000	0.731	-
	CAI	1.000	0.731	0.376	1.000
